# 9a-Hy­droxy-3,8a-dimethyl-5-methyl­ene-4,4a,5,6,9,9a-hexa­hydro­naphtho­[2,3-*b*]furan-2(8a*H*)-one

**DOI:** 10.1107/S1600536811006519

**Published:** 2011-02-26

**Authors:** Wen-Hong Liu, Jie He, Zhi-Shan Ding, Zhong-Cheng Song, Zha-Jun Zhan

**Affiliations:** a Bioengineering Department, Zhejiang Traditional Chinese Medicine University, Hangzhou 310053, People’s Republic of China; b Department of Life Science, Zhejiang Traditional Chinese Medicine University, Hangzhou 310053, People’s Republic of China; c College of Pharmaceutical Science, Zhejiang University of Technology, Hangzhou 310032, People’s Republic of China

## Abstract

The title compound, C_15_H_18_O_3_, was isolated from *Lacta­rius piperatus* (Fr.) S. F. Gary collected from the Kunming area in Yunnan province, China. The central cyclo­hexyl ring adopts a chair conformation, while the furan­one ring is close to planar (r.m.s. deviation = 0.0174 Å). The remaining methyl­ene cyclo­hexene ring has a flattened chair conformation. In the crystal, mol­ecules are linked *via* inter­molecular O—H⋯O and C—H⋯O hydrogen bonds into zigzag chains along the *a* axis.

## Related literature

For the distribution of the fungus *Lacta­rius piperatus* in China, see: Xie *et al.* (1996 ▶). For the anti-tumor activity of this species see: Mo *et al.* (1995 ▶). A series of sesquiterpenes has been isolated from the genus *Lacta­rius*, see: De Bernardi *et al.* (1993 ▶); Sterner *et al.* (1990 ▶). For the isolation of amino acids and sesquiterpenes from *L. piperatus* growing in Europe and Japan and their biological activity, see: Fushiya *et al.* (1988 ▶); Sterner *et al.* (1985*a* ▶,*b* ▶); Yaoita *et al.* (1999 ▶). For standard bond lengths, see: Allen *et al.* (1987 ▶).
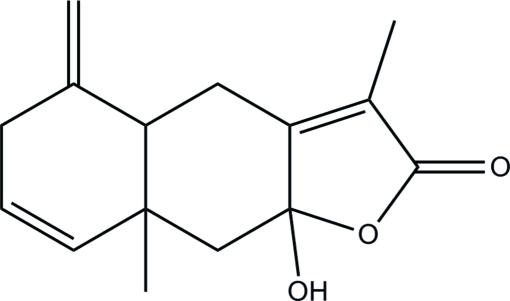


## Experimental

### 

#### Crystal data

C_15_H_18_O_3_
*M* *_r_* = 247.30
Orthorhombic, 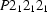

*a* = 9.5150 (19) Å
*b* = 10.885 (2) Å
*c* = 12.594 (3) Å
*V* = 1304.4 (5) Å^3^
*Z* = 4
Mo *K*α radiation
μ = 0.09 mm^−1^
*T* = 298 K
0.30 × 0.20 × 0.20 mm

#### Data collection

Enraf–Nonius CAD-4 diffractometer
Absorption correction: ψ scan (North *et al.*, 1968 ▶) *T*_min_ = 0.975, *T*_max_ = 0.983
2616 measured reflections
1367 independent reflections
1209 reflections with *I* > 2σ(*I*)
*R*_int_ = 0.021
3 standard reflections every 200 reflections  intensity decay: 1%

#### Refinement

*R*[*F* ^2^ > 2σ(*F* ^2^)] = 0.035
*wR*(*F* ^2^) = 0.091
*S* = 1.06
1367 reflections
164 parameters
H-atom parameters constrained
Δρ_max_ = 0.15 e Å^−3^
Δρ_min_ = −0.12 e Å^−3^

### 

Data collection: *CAD-4 Software* (Enraf–Nonius, 1989 ▶); cell refinement: *CAD-4 Software*; data reduction: *XCAD4* (Harms & Wocadlo, 1995 ▶); program(s) used to solve structure: *SHELXS97* (Sheldrick, 2008 ▶); program(s) used to refine structure: *SHELXL97* (Sheldrick, 2008 ▶); molecular graphics: *SHELXTL* (Sheldrick, 2008 ▶); software used to prepare material for publication: *SHELXL97*.

## Supplementary Material

Crystal structure: contains datablocks global, I. DOI: 10.1107/S1600536811006519/sj5106sup1.cif

Structure factors: contains datablocks I. DOI: 10.1107/S1600536811006519/sj5106Isup2.hkl

Additional supplementary materials:  crystallographic information; 3D view; checkCIF report

## Figures and Tables

**Table 1:** Hydrogen-bond geometry (Å, °)

*D*—H⋯*A*	*D*—H	H⋯*A*	*D*⋯*A*	*D*—H⋯*A*
O3—H3*A*⋯O2^i^	0.82	1.99	2.796 (2)	169
C1—H1*A*⋯O1	0.93	2.63	3.495 (2)	118

Symmetry code: (i) 
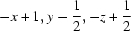
.

**Fig. 1.**
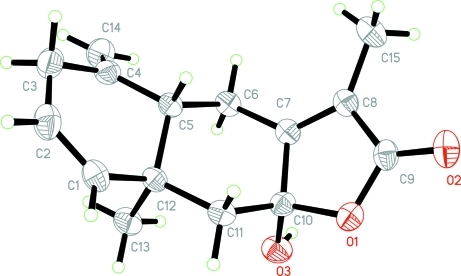
The molecular structure of the title compounds with atom labels and the 30% probability displacement ellipsoids for non-hydrogen atoms.

**Fig. 2.**
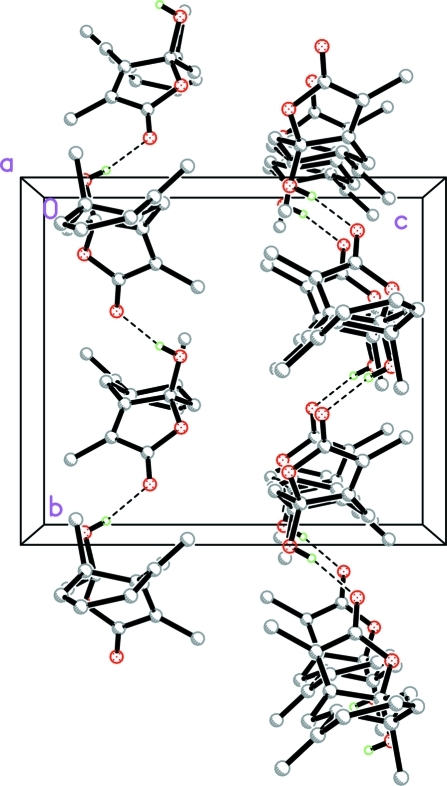
Molecular packing of the title compound, viewed along the a axis. Hydrogen bonds are drawn as dashed lines.

## supplementary crystallographic information

### Comment

The fungus *Lactarius piperatus* (Fr.) S. F. Gary (family Russulaceae, Basidiomycotina) is widely distributed in China (Xie *et al.*, 1996). The ethanolic extract has been reported to inhibit the growth of several tumor cell lines (Mo *et al.*, 1995). *L. Piperatus*, growing in Europe and Japan, has been investigated, and a new amino acid and a few sesquiterpenes were isolated (Fushiya *et al.*, 1988; Sterner *et al.*, 1985*a*; Yaoita *et al.*, 1999), but *L. piperatus* growing in China has not been previously investigated chemically. A series of sesquiterpenes, belonging to the marasmane, lactarane, isolactarane, and secolactarane types, has been isolated from the genus of Lactarius (De Bernardi *et al.*, 1993, Sterner *et al.*, 1990). These sesquiterpenes provide a chemical defense system against parasites and predators (Sterner *et al.*, 1985*b*).

The molecular structure of the title compound is shown in Fig. 1. All bond lengths are within normal ranges (Allen *et al.*, 1987). The central cyclohexyl ring C5-C6-C7-C10-C11-C12 adopts a chair conformation. The dihedral angle between the C7-C8-C9-O1-C10 ring and the plane defined by C12-C1-C2-C3-C4 is 75.3 (3)°. Atom C5 deviates 0.692 (2) Å from the plane defined by C12-C1-C2-C3-C4. In the crystal, molecules are linked via intermolecular O—H···O hydrogen bonds (Table 1) to form chains along the *a* axis (Fig. 2).

### Experimental

Plant material: *Lactarius piperatus* (Fr.) S. F. Gary was collected from the Kunming area in Yunnan province of China and authenticated by Prof. Mu Zang, Kunming  Institute of Botany, where a voucher specimen labeled as HKAS 30213, was deposited. Extraction and Isolation: The fresh mushroom (5 kg) was extracted with 95% EtOH and yielded 91 g of crude extract, which was then suspended in 2 L water. The suspension was partitioned with EtOAc (4× 200 ml) to give an EtOAc-soluble portion, and a water-soluble fraction. After removal of the EtOAc under reduced pressure, 49 g of dark residue was obtained, and this was subjected to silica-gel chromatography, eluted with a stepwise gradient solvent system of petroleum/acetone 10 : 0 to 5 : 5, followed by MeOH, to afford four major fractions (monitored by TLC). Fr. 1 consisted mainly of fatty acids. Fr. 4 was much smaller and complex. The separation and purification were focused on Fr. 2 and 3, in which the sesquiterpenes were concentrated. A portion of sub-fraction Fr. 2 was re-chromatographed on silica gel using a petroleum ether-acetone (8:2) system and the isolated product was recrystallized from chloroform-methanol (7:3) to yield the active component as light colorless crystals.

### Refinement

H atoms were positioned geometrically and refined using the riding-model approximation, with C—H = 0.93–0.97 Å, O—H = 0.82 Å, and *U*_iso_(H) = 1.2*U*_eq_(C) or *U*_iso_(H) = 1.5*U*_eq_(O). In the absence of significant anomalous dispersion effects, Freidel pairs were merged.

### Crystal data

**Table:**

C_15_H_18_O_3_	*F*(000) = 532
*M**_r_* = 247.30	*D*_x_ = 1.259 Mg m^−^^3^
Orthorhombic, *P*2_1_2_1_2_1_	Mo *K*α radiation, λ = 0.71073 Å
Hall symbol: P 2ac 2ab	Cell parameters from 25 reflections
*a* = 9.5150 (19) Å	θ = 10–13°
*b* = 10.885 (2) Å	µ = 0.09 mm^−^^1^
*c* = 12.594 (3) Å	*T* = 298 K
*V* = 1304.4 (5) Å^3^	Block, colourless
*Z* = 4	0.30 × 0.20 × 0.20 mm

### Data collection

**Table:**

Enraf–Nonius CAD-4 diffractometer	1209 reflections with *I* > 2σ(*I*)
Radiation source: fine-focus sealed tube	*R*_int_ = 0.021
graphite	θ_max_ = 25.3°, θ_min_ = 2.5°
ω/2θ scans	*h* = 0→11
Absorption correction: ψ scan (North *et al.*, 1968)	*k* = 0→12
*T*_min_ = 0.975, *T*_max_ = 0.983	*l* = −15→15
2616 measured reflections	3 standard reflections every 200 reflections
1367 independent reflections	intensity decay: 1%

### Refinement

**Table:**

Refinement on *F*^2^	Secondary atom site location: difference Fourier map
Least-squares matrix: full	Hydrogen site location: inferred from neighbouring sites
*R*[*F*^2^ > 2σ(*F*^2^)] = 0.035	H-atom parameters constrained
*wR*(*F*^2^) = 0.091	*w* = 1/[σ^2^(*F*_o_^2^) + (0.0474*P*)^2^ + 0.1815*P*] where *P* = (*F*_o_^2^ + 2*F*_c_^2^)/3
*S* = 1.06	(Δ/σ)_max_ = 0.001
1367 reflections	Δρ_max_ = 0.15 e Å^−^^3^
164 parameters	Δρ_min_ = −0.12 e Å^−^^3^
0 restraints	Extinction correction: *SHELXL97* (Sheldrick, 2008), Fc^*^=kFc[1+0.001xFc^2^λ^3^/sin(2θ)]^-1/4^
Primary atom site location: structure-invariant direct methods	Extinction coefficient: 0.036 (3)

### Special details

**Table:**

Geometry. All e.s.d.'s (except the e.s.d. in the dihedral angle between two l.s. planes) are estimated using the full covariance matrix. The cell e.s.d.'s are taken into account individually in the estimation of e.s.d.'s in distances, angles and torsion angles; correlations between e.s.d.'s in cell parameters are only used when they are defined by crystal symmetry. An approximate (isotropic) treatment of cell e.s.d.'s is used for estimating e.s.d.'s involving l.s. planes.
Refinement. Refinement of *F*^2^ against ALL reflections. The weighted *R*-factor *wR* and goodness of fit *S* are based on *F*^2^, conventional *R*-factors *R* are based on *F*, with *F* set to zero for negative *F*^2^. The threshold expression of *F*^2^ > σ(*F*^2^) is used only for calculating *R*-factors(gt) *etc*. and is not relevant to the choice of reflections for refinement. *R*-factors based on *F*^2^ are statistically about twice as large as those based on *F*, and *R*- factors based on ALL data will be even larger.

### Fractional atomic coordinates and isotropic or equivalent isotropic displacement parameters (Å^2^)

**Table:**

	*x*	*y*	*z*	*U*_iso_*/*U*_eq_	
O1	0.40511 (17)	0.69401 (16)	0.36817 (13)	0.0514 (5)	
C1	−0.0968 (3)	0.6294 (2)	0.4001 (2)	0.0512 (6)	
H1A	−0.0918	0.6506	0.4715	0.061*	
O2	0.51038 (18)	0.85545 (16)	0.29192 (14)	0.0627 (5)	
C2	−0.2158 (3)	0.6509 (3)	0.3493 (2)	0.0609 (8)	
H2A	−0.2893	0.6871	0.3866	0.073*	
O3	0.34477 (19)	0.48626 (15)	0.36469 (14)	0.0576 (5)	
H3A	0.3854	0.4559	0.3136	0.086*	
C3	−0.2383 (3)	0.6201 (3)	0.2348 (2)	0.0607 (8)	
H3B	−0.2599	0.6949	0.1962	0.073*	
H3C	−0.3188	0.5658	0.2288	0.073*	
C4	−0.1128 (3)	0.5590 (2)	0.18373 (19)	0.0478 (6)	
C5	0.0243 (2)	0.60217 (19)	0.22901 (16)	0.0387 (5)	
H5A	0.0223	0.6920	0.2238	0.046*	
C6	0.1574 (3)	0.5615 (2)	0.16947 (18)	0.0440 (6)	
H6A	0.1696	0.4732	0.1748	0.053*	
H6B	0.1513	0.5837	0.0950	0.053*	
C7	0.2764 (2)	0.6267 (2)	0.22127 (18)	0.0405 (5)	
C8	0.3544 (2)	0.7232 (2)	0.19078 (17)	0.0424 (5)	
C9	0.4327 (2)	0.7661 (2)	0.2838 (2)	0.0460 (6)	
C10	0.2974 (3)	0.6033 (2)	0.33811 (19)	0.0425 (6)	
C11	0.1634 (2)	0.6320 (2)	0.39810 (17)	0.0423 (5)	
H11A	0.1507	0.7204	0.3999	0.051*	
H11B	0.1735	0.6039	0.4708	0.051*	
C12	0.0308 (2)	0.5729 (2)	0.34989 (18)	0.0395 (5)	
C13	0.0256 (3)	0.4326 (2)	0.3715 (2)	0.0569 (7)	
H13B	0.0301	0.4181	0.4466	0.085*	
H13C	0.1040	0.3934	0.3374	0.085*	
H13D	−0.0604	0.3994	0.3439	0.085*	
C14	−0.1255 (3)	0.4774 (3)	0.1063 (2)	0.0644 (8)	
H14A	−0.0457	0.4425	0.0761	0.077*	
H14B	−0.2142	0.4549	0.0821	0.077*	
C15	0.3624 (3)	0.7890 (3)	0.0874 (2)	0.0607 (7)	
H15A	0.3043	0.7478	0.0363	0.091*	
H15B	0.4580	0.7895	0.0629	0.091*	
H15C	0.3303	0.8719	0.0964	0.091*	

### Atomic displacement parameters (Å^2^)

**Table:**

	*U*^11^	*U*^22^	*U*^33^	*U*^12^	*U*^13^	*U*^23^
O1	0.0459 (9)	0.0572 (10)	0.0510 (9)	−0.0036 (9)	−0.0053 (8)	−0.0002 (9)
C1	0.0518 (14)	0.0515 (14)	0.0503 (13)	−0.0019 (13)	0.0146 (12)	−0.0027 (12)
O2	0.0517 (10)	0.0567 (11)	0.0797 (12)	−0.0136 (10)	0.0040 (11)	−0.0053 (10)
C2	0.0462 (15)	0.0611 (17)	0.0754 (19)	0.0031 (14)	0.0136 (14)	−0.0024 (16)
O3	0.0614 (11)	0.0476 (9)	0.0636 (11)	0.0159 (9)	0.0033 (10)	0.0107 (9)
C3	0.0448 (14)	0.0588 (16)	0.0784 (19)	−0.0034 (13)	−0.0039 (13)	0.0088 (16)
C4	0.0525 (15)	0.0402 (12)	0.0507 (13)	−0.0079 (12)	−0.0067 (12)	0.0077 (11)
C5	0.0455 (13)	0.0272 (10)	0.0433 (13)	−0.0020 (10)	0.0012 (11)	0.0012 (9)
C6	0.0532 (14)	0.0388 (12)	0.0402 (11)	−0.0012 (12)	0.0036 (11)	−0.0045 (10)
C7	0.0425 (12)	0.0366 (12)	0.0425 (11)	0.0061 (10)	0.0074 (10)	−0.0040 (11)
C8	0.0407 (12)	0.0394 (11)	0.0471 (12)	0.0056 (11)	0.0090 (10)	−0.0018 (10)
C9	0.0358 (11)	0.0439 (13)	0.0582 (14)	0.0033 (11)	0.0074 (11)	−0.0032 (12)
C10	0.0436 (12)	0.0370 (12)	0.0470 (12)	0.0028 (11)	−0.0037 (11)	0.0014 (10)
C11	0.0512 (13)	0.0365 (11)	0.0391 (11)	0.0019 (11)	0.0017 (11)	−0.0005 (10)
C12	0.0454 (13)	0.0328 (11)	0.0403 (11)	−0.0010 (10)	0.0039 (10)	0.0016 (10)
C13	0.0671 (17)	0.0367 (12)	0.0668 (16)	−0.0045 (13)	0.0016 (15)	0.0124 (12)
C14	0.0676 (18)	0.0618 (16)	0.0638 (16)	−0.0190 (15)	−0.0125 (15)	−0.0062 (14)
C15	0.0710 (18)	0.0546 (15)	0.0566 (14)	−0.0034 (15)	0.0161 (14)	0.0059 (13)

### Geometric parameters (Å, °)

**Table:**

O1—C9	1.347 (3)	C6—H6A	0.9700
O1—C10	1.473 (3)	C6—H6B	0.9700
C1—C2	1.321 (4)	C7—C8	1.342 (3)
C1—C12	1.501 (3)	C7—C10	1.507 (3)
C1—H1A	0.9300	C8—C9	1.465 (3)
O2—C9	1.225 (3)	C8—C15	1.487 (3)
C2—C3	1.496 (4)	C10—C11	1.515 (3)
C2—H2A	0.9300	C11—C12	1.541 (3)
O3—C10	1.392 (3)	C11—H11A	0.9700
O3—H3A	0.8200	C11—H11B	0.9700
C3—C4	1.510 (4)	C12—C13	1.552 (3)
C3—H3B	0.9700	C13—H13B	0.9600
C3—H3C	0.9700	C13—H13C	0.9600
C4—C14	1.325 (4)	C13—H13D	0.9600
C4—C5	1.499 (3)	C14—H14A	0.9300
C5—C6	1.537 (3)	C14—H14B	0.9300
C5—C12	1.557 (3)	C15—H15A	0.9600
C5—H5A	0.9800	C15—H15B	0.9600
C6—C7	1.488 (3)	C15—H15C	0.9600
			
C9—O1—C10	108.89 (17)	O2—C9—C8	128.8 (2)
C2—C1—C12	124.2 (2)	O1—C9—C8	110.22 (19)
C2—C1—H1A	117.9	O3—C10—O1	109.03 (18)
C12—C1—H1A	117.9	O3—C10—C7	115.6 (2)
C1—C2—C3	123.3 (3)	O1—C10—C7	103.28 (19)
C1—C2—H2A	118.3	O3—C10—C11	110.0 (2)
C3—C2—H2A	118.3	O1—C10—C11	108.62 (18)
C10—O3—H3A	109.5	C7—C10—C11	109.95 (19)
C2—C3—C4	113.4 (2)	C10—C11—C12	113.98 (17)
C2—C3—H3B	108.9	C10—C11—H11A	108.8
C4—C3—H3B	108.9	C12—C11—H11A	108.8
C2—C3—H3C	108.9	C10—C11—H11B	108.8
C4—C3—H3C	108.9	C12—C11—H11B	108.8
H3B—C3—H3C	107.7	H11A—C11—H11B	107.7
C14—C4—C5	124.7 (2)	C1—C12—C11	108.96 (18)
C14—C4—C3	122.4 (2)	C1—C12—C13	107.7 (2)
C5—C4—C3	112.8 (2)	C11—C12—C13	111.6 (2)
C4—C5—C6	116.17 (18)	C1—C12—C5	107.21 (19)
C4—C5—C12	110.03 (18)	C11—C12—C5	109.40 (18)
C6—C5—C12	112.67 (19)	C13—C12—C5	111.8 (2)
C4—C5—H5A	105.7	C12—C13—H13B	109.5
C6—C5—H5A	105.7	C12—C13—H13C	109.5
C12—C5—H5A	105.7	H13B—C13—H13C	109.5
C7—C6—C5	106.03 (17)	C12—C13—H13D	109.5
C7—C6—H6A	110.5	H13B—C13—H13D	109.5
C5—C6—H6A	110.5	H13C—C13—H13D	109.5
C7—C6—H6B	110.5	C4—C14—H14A	120.0
C5—C6—H6B	110.5	C4—C14—H14B	120.0
H6A—C6—H6B	108.7	H14A—C14—H14B	120.0
C8—C7—C6	132.0 (2)	C8—C15—H15A	109.5
C8—C7—C10	109.8 (2)	C8—C15—H15B	109.5
C6—C7—C10	116.6 (2)	H15A—C15—H15B	109.5
C7—C8—C9	107.6 (2)	C8—C15—H15C	109.5
C7—C8—C15	131.0 (2)	H15A—C15—H15C	109.5
C9—C8—C15	121.3 (2)	H15B—C15—H15C	109.5
O2—C9—O1	120.9 (2)		
			
C12—C1—C2—C3	0.7 (5)	C9—O1—C10—C7	4.8 (2)
C1—C2—C3—C4	1.4 (4)	C9—O1—C10—C11	−111.9 (2)
C2—C3—C4—C14	148.5 (3)	C8—C7—C10—O3	−122.8 (2)
C2—C3—C4—C5	−32.3 (3)	C6—C7—C10—O3	69.8 (3)
C14—C4—C5—C6	9.1 (3)	C8—C7—C10—O1	−3.8 (2)
C3—C4—C5—C6	−170.1 (2)	C6—C7—C10—O1	−171.18 (18)
C14—C4—C5—C12	−120.4 (3)	C8—C7—C10—C11	111.9 (2)
C3—C4—C5—C12	60.4 (3)	C6—C7—C10—C11	−55.4 (3)
C4—C5—C6—C7	173.61 (19)	O3—C10—C11—C12	−79.2 (2)
C12—C5—C6—C7	−58.2 (2)	O1—C10—C11—C12	161.56 (18)
C5—C6—C7—C8	−105.0 (3)	C7—C10—C11—C12	49.2 (3)
C5—C6—C7—C10	59.0 (2)	C2—C1—C12—C11	144.3 (3)
C6—C7—C8—C9	166.2 (2)	C2—C1—C12—C13	−94.5 (3)
C10—C7—C8—C9	1.5 (2)	C2—C1—C12—C5	26.0 (3)
C6—C7—C8—C15	−9.7 (4)	C10—C11—C12—C1	−167.5 (2)
C10—C7—C8—C15	−174.5 (2)	C10—C11—C12—C13	73.7 (3)
C10—O1—C9—O2	173.7 (2)	C10—C11—C12—C5	−50.6 (2)
C10—O1—C9—C8	−4.2 (2)	C4—C5—C12—C1	−54.9 (2)
C7—C8—C9—O2	−176.0 (2)	C6—C5—C12—C1	173.76 (18)
C15—C8—C9—O2	0.4 (4)	C4—C5—C12—C11	−172.89 (18)
C7—C8—C9—O1	1.7 (3)	C6—C5—C12—C11	55.7 (2)
C15—C8—C9—O1	178.1 (2)	C4—C5—C12—C13	63.0 (3)
C9—O1—C10—O3	128.3 (2)	C6—C5—C12—C13	−68.4 (3)

### Hydrogen-bond geometry (Å, °)

**Table:**

*D*—H···*A*	*D*—H	H···*A*	*D*···*A*	*D*—H···*A*
O3—H3A···O2^i^	0.82	1.99	2.796 (2)	169
C1—H1A···O1^ii^	0.93	2.63	3.495 (2)	118

Symmetry codes: (i) −*x*+1, *y*−1/2, −*z*+1/2; (ii) .
